# Case Report: Fatal pneumonitis caused by Camrelizumab and Erlotinib in a patient with metastatic pancreatic cancer

**DOI:** 10.3389/fonc.2026.1746639

**Published:** 2026-06-10

**Authors:** Yanyan Jiang, Lihong Ma, Bei Lu, Wei Tian

**Affiliations:** 1 Department of Respiratory and Critical Care Medicine, Zibo Central Hospital, Shandong University, Zibo, Shandong, China; 2 Department of Gastroenterology, Zibo Central Hospital, Shandong University, Zibo, Shandong, China; 3 Department of Nuclear Medicine and Radiotherapy, Zibo Central Hospital, Shandong University, Zibo, Shandong, China; 4 Department of Oncology, Zibo Central Hospital, Shandong University, Zibo, Shandong, China

**Keywords:** case report, Erlotinib, immune checkpoint inhibitors, pancreatic cancer, pneumonitis

## Abstract

Pancreatic cancer continues to be one of the foremost contributors to cancer-related mortality. Erlotinib is a targeted agent that has shown a significant improvement in outcomes for advanced pancreatic cancer when administered alongside chemotherapy. Recent small-scale clinical studies have indicated promising results for the use of immune checkpoint inhibitors in the management of advanced pancreatic cancer. We present a case involving a 70-year-old male patient who presented with abdominal pain and a subcutaneous nodular lesion located in the frontal region. The patient was ultimately diagnosed with metastatic pancreatic cancer and was refractory to gemcitabine and nab-paclitaxel. Following treatment with Camrelizumab, Erlotinib and chemotherapy in the second-line setting, the patient developed fatal pneumonitis. Despite receiving optimal treatment with steroid and antibiotic therapy, the patient ultimately succumbed to respiratory failure. This case demonstrates that the combination of immunotherapy and Erlotinib may not constitute a safe therapeutic strategy for patients with advanced pancreatic cancer, as it can lead to severe pneumonitis. Significant caution should also be exercised when administering Erlotinib to patients who have recently undergone immune checkpoint inhibitor therapy.

## 1 Introduction

In 2020, pancreatic cancer ranked as the seventh leading cause of cancer-related deaths, with an estimated 496,000 new cases and 466,000 fatalities (1). The introduction of multidrug chemotherapy regimens, including fluorouracil, leucovorin, irinotecan, and oxaliplatin (collectively referred to as FOLFIRINOX), as well as nab-paclitaxel in combination with gemcitabine (known as AG), has led to significant improvements in overall survival (OS) for patients with advanced and metastatic pancreatic cancer (2, 3).

Most guidelines recommend fluorouracil-based therapy in the second-line setting for patients with gemcitabine-refractory advanced pancreatic cancer. The NAPOLI-1 study has demonstrated that the combination of nanoliposomal irinotecan, fluorouracil, and folinic acid significantly prolongs OS in patients with gemcitabine-refractory advanced pancreatic cancer (4). Furthermore, a Phase III trial has shown statistically significant improvements in OS for pancreatic cancer patients when Erlotinib, an epidermal growth factor receptor kinase inhibitor (EGFR-TKI), is added to chemotherapy (5). Recent small-scale clinical studies have demonstrated promising results regarding the use of anti-Programmed Death Receptor 1 (PD-1) antibodies in combination with chemotherapy and targeted therapy for managing advanced pancreatic cancer (6, 7). Yuanbo Bi et al. reported a case of a patient with locally advanced pancreatic cancer who received treatment with Camrelizumab, a PD-1 inhibitor, in conjunction with chemotherapy. This treatment approach resulted in a favorable prognosis for the patient (8). This case underscores the potential of immunotherapy as a viable option for the treatment of pancreatic cancer. In contrast, we present a patient with metastatic pancreatic cancer who developed fatal pneumonitis following treatment with Camrelizumab, Erlotinib, and chemotherapy.

## 2 Case presentation

A 70-year-old male with no history of smoking (Eastern Cooperative Oncology Group performance status 1) presented with a one-month history of intermittent upper abdominal pain and a subcutaneous nodular lesion located in the frontal region (**Figure 1A**). The patient reported no significant past medical history, including pulmonary diseases, and denied any occupational exposure to gases, dust, or fumes. He received inactivated coronavirus disease 2019 (COVID-19) vaccines in 2020 and had not received any other vaccinations since then. Furthermore, he had not undergone any interventions for his symptoms. An enhanced computed tomography (CT) scan revealed a space-occupying lesion at the body of the pancreas (**Figure 1B**), accompanied by multiple enlarged lymph nodes in the retroperitoneum (**Figure 1C**). The chest CT examination identified multiple nodular lesions in both lungs, suggestive of metastatic disease (**Figures 1D–F**). Additionally, serum tumor marker levels were significantly elevated, with carcinoembryonic antigen (CEA) measuring 54 ng/mL (reference range: 0–5 ng/mL) and cancer antigen 19-9 (CA 19-9) measuring 22,251 U/mL (reference range: 0–35 U/mL). These findings raised a strong suspicion of metastatic pancreatic cancer.

**Figure 1:**
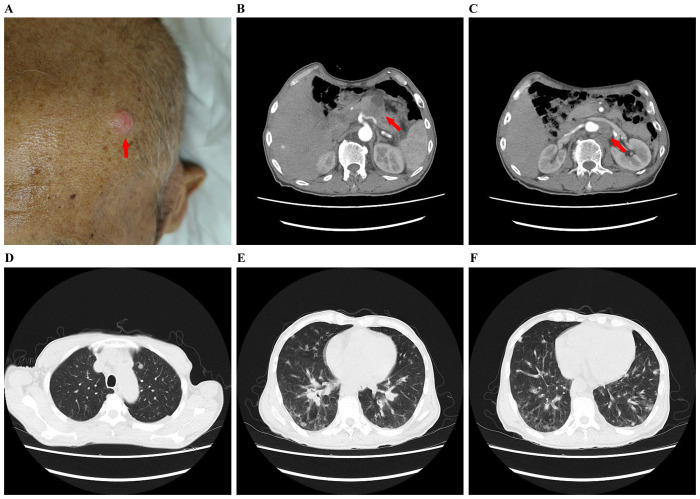
Symptom and radiological findings. **(A)** The subcutaneous nodular lesion located in the frontal region. **(B)** CT scan revealed a space-occupying lesion at the body of the pancreas. **(C)** CT scan showed enlarged lymph nodes in the retroperitoneum. **(D–F)** CT scan identified multiple nodular lesions in both lungs.

### 2.1 Auxiliary examinations

A puncture biopsy was performed on the subcutaneous nodular lesion in the frontal region. The pathological analysis revealed poorly differentiated adenocarcinoma. The immunohistochemical results were as follows: CDX2 (partially +), CK7 (+), MLH-1 (+), PMS-1 (+), MSH-2 (+), MSH-6 (+), Ki-67 (+, 5%) (**Figure 2**). By correlating the imaging findings, tumor markers, and pathological results, a diagnosis of metastatic pancreatic cancer was established.

**Figure 2:**
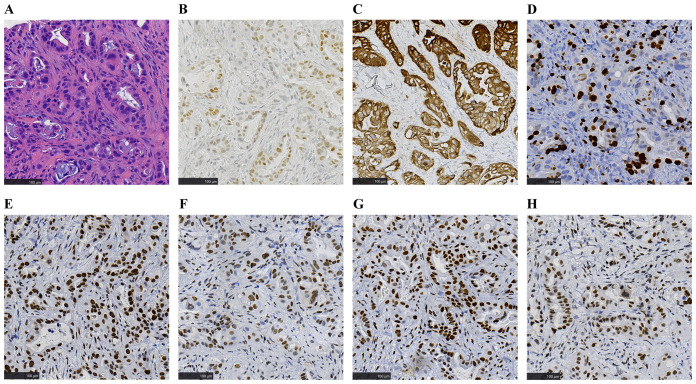
Histological findings. **(A)** Hematoxylin and eosin staining of the nodular lesion located in the frontal region showed moderately differentiated adenocarcinoma (×200). **(B)** Immunohistochemical staining for CDX-2 showed partial positivity. **(C)** Immunohistochemical staining for CK-7 showed strong positivity. **(D)** Immunohistochemical staining for Ki-67 showed positivity for 5%. **(E–H)** Immunohistochemical staining for MLH-1, MSH-2, MSH-6 and PMS-2 showed strong positivity. Scale bar=100 μm.

### 2.2 First-line treatment

The patient initially received a chemotherapy regimen referred to as AG, which consisted of nab-paclitaxel and gemcitabine. The treatment protocol involved administering nab-paclitaxel at a dosage of 200 mg on days 1 and 8, in conjunction with gemcitabine at a dosage of 1.6 g on days 2 and 9, with cycles repeated every 21 days for a total of two cycles. Follow-up CT scans of the abdomen and lungs revealed a stable mass in the body of the pancreas and stable lymph nodes in the retroperitoneum, along with enlarged nodular lesions in the lungs (**Figure 3**). Serum CA 19–9 levels continued to rise, indicating resistance to the initial chemotherapy.

**Figure 3:**
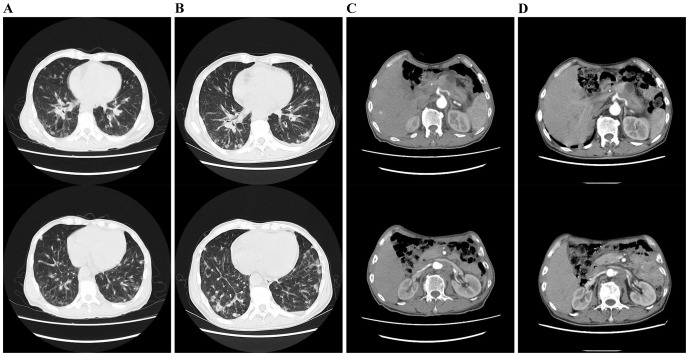
Chest and abdominal CT scan findings during the first-line chemotherapy treatment. **(A)** CT scan of the lung prior to treatment. **(B)** CT scan of the lung after two cycles of nab-paclitaxel and gemcitabine chemotherapy revealed enlarged nodular lesions in the lungs. **(C)** CT scan of the abdomen prior to treatment. **(D)** CT scan of the abdomen after two cycles of ab-paclitaxel and gemcitabine chemotherapy revealed a stable mass in the pancreas and stable lymph nodes in the retroperitoneum.

### 2.3 Second-line treatment

The second-line chemotherapy regimen was modified to FOLFIRI, which consists of irinotecan administered intravenously at a dose of 290 mg on day 1, a continuous infusion of 5-FU at 3,750 mg over 46 hours, an additional intravenous dose of 500 mg of 5-FU on day 1, and leucovorin (LV) at a dose of 650 mg administered intravenously on day 1, given biweekly. This regimen was further supplemented by the PD-1 inhibitor Camrelizumab, administered at a dose of 200 mg every 21 days, along with Erlotinib at a daily dose of 150 mg.

### 2.4 Severe pneumonitis

Upon admission (day 20 of Camrelizumab therapy), the patient reported a four-day history of progressively worsening shortness of breath and severe fatigue. He denied experiencing any fevers, chills, or exposure to individuals with infectious diseases. His oxygen saturation (SpO_2_) was recorded at 89% while on room air. An arterial blood gas (ABG) analysis revealed a ratio of arterial oxygen tension (PaO_2_) to fractional inspired oxygen (FiO_2_) of 180 mm Hg. Laboratory investigations revealed systemic inflammation and neutrophilia, characterized by a white blood cell count of 15.3×10^9^/L, with neutrophils constituting 83.2% of the total. Additionally, the C-reactive protein level was found to be elevated at 89 mg/L, while serum albumin level decreased to 31 g/L.

A chest CT scan revealed diffuse ground-glass opacities throughout the lungs (**Figure 4B**). The viral respiratory pathogen panel and sputum cultures yielded negative results. Additionally, the fungal Polymerase Chain Reaction (PCR) detection of sputum, specifically targeting aspergillus, cryptococcus neoformans, and *pneumocystis jirovecii*, also returned negative results. Consequently, the patient was diagnosed with grade 4 anti-PD-1-induced pneumonitis.

**Figure 4:**
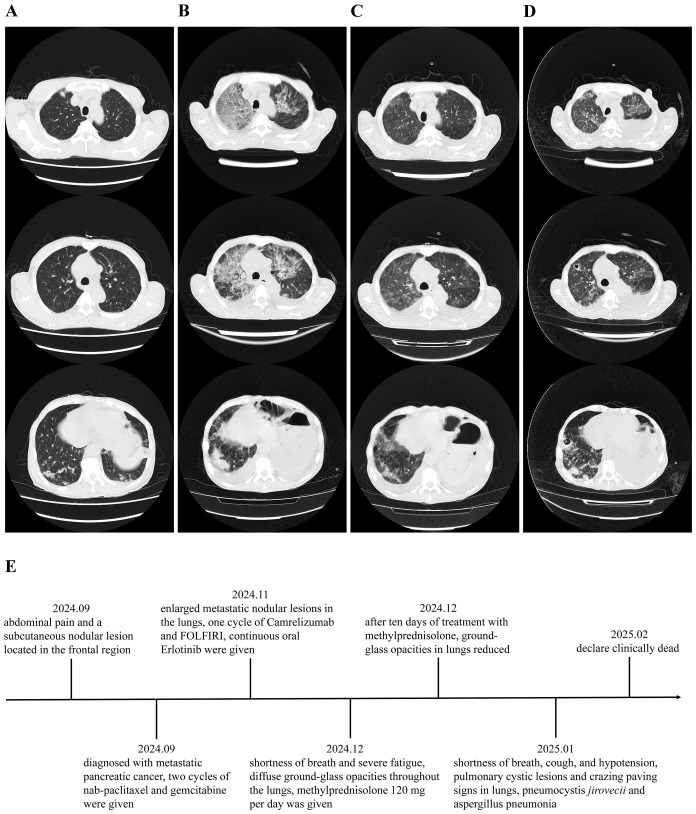
Chest CT scan of severe pneumonitis and timeline of the case. **(A)** CT scan of the lung prior to treatment. **(B)** CT scan of the lung revealed grade four anti-PD-1-induced pneumonitis after one cycle of Camrelizumab, Erlotinib and FOLFIRI. **(C)** CT scan of the lung revealed a reduction in pneumonitis after the treatment of methylprednisolone for ten days. **(D)** CT scan of the lung revealed newly developed pulmonary cystic lesions and crazing paving signs during the tapering of methylprednisolone. **(E)** Timeline of the clinical case presented.

Immunosuppressive treatment with methylprednisolone was initiated at a dosage of 120 mg per day, leading to an improvement in clinical symptoms. After ten days, a chest CT scan revealed a reduction in ground-glass opacities (**Figure 4C**). Additionally, the ratio of PaO_2_ to FiO_2_ increased to 310 mm Hg. The white blood cell count and the C-reactive protein level decreased to 9.6×10^9^/L and 23 mg/L, respectively. Subsequently, the dosage of methylprednisolone was tapered to 80 mg, continuing for an additional ten days. Five days after the dosage was reduced to 40 mg, the patient exhibited significant shortness of breath, a nonproductive cough, and hypotension. A chest CT scan revealed pulmonary cystic lesions and crazing paving signs (**Figure 4D**). These findings were characteristic of *pneumocystis jirovecii* pneumonia (PJP). The PCR detection of fungal in sputum samples for *pneumocystis jirovecii* and aspergillus yielded positive results.

Subsequently, the patient was treated with *Voriconazole* and *Trimethoprim*/*Sulfamethoxazole* (TMP/SMX). Despite appropriate antibiotic therapy and intensive treatment, the patient succumbed to respiratory failure two weeks later. A timeline of the clinical case was presented (**Figure 4E**).

## 3 Discussion

Patients with advanced pancreatic cancer who are resistant to first-line chemotherapy face an extremely poor prognosis. Consequently, second-line treatments involving immunotherapy or erlotinib combined with other chemotherapy regimens may offer potential benefits. However, the safety of concurrently administering immunotherapy, Erlotinib, and chemotherapy has not been previously reported. In this report, we present a case of severe pneumonitis that developed following treatment with Camrelizumab, Erlotinib, and chemotherapy. The patient required hospitalization and subsequent steroid therapy, which resulted in a promising response. However, the patient developed PJP and aspergillus pneumonia during corticosteroid taper and subsequently died. This case report underscores the potential risks associated with the simultaneous use of immune checkpoint inhibitor and Erlotinib in patients with advanced pancreatic cancer.

Immunotherapy can lead to immune-related adverse events, one of which is pneumonitis. Although typically mild, pneumonitis can also be potentially life-threatening. Approximately 3% of patients receiving anti-PD-1 or anti-Programmed Cell Death Ligand 1 (PD-L1) monotherapy develop pneumonitis. Although this condition is typically mild, about 1% of patients may experience severe pneumonitis, classified as grade 3 or 4 (9). The diagnosis of pneumonitis associated with immunotherapy requires the exclusion of infections, cancer progression, and radiation-induced pneumonia. Notably, this patient exhibited no signs of tumor progression or infection at the time of pneumonitis diagnosis.

It is crucial to initiate an immune-mediated tumor response in patients with advanced pancreatic cancer, and this area of research warrants further investigation. While both immunotherapy and Erlotinib show promise in treating pancreatic cancer, the combination of these two therapies may not represent a safe therapeutic strategy for patients. The combination of Osimertinib, a third-generation EGFR-TKI, with Durvalumab, a PD-L1 inhibitor, significantly increased the incidence of pneumonitis, as demonstrated in the TATTON trial designed for non-small cell lung cancer (NSCLC). While the occurrence of pneumonitis was relatively low with Osimertinib alone (2.9%) and with Durvalumab alone (2%), the concurrent administration observed in this study resulted in an incidence of 38% (10).

Furthermore, the safety of sequential immunotherapy and Erlotinib in pancreatic cancer remains a critical concern. In NSCLC, immunotherapy followed by osimertinib is associated with severe immune-related adverse events, which are most frequently observed in patients who have recently received anti-PD-1 or anti-PD-L1 therapy. Notably, no adverse events were reported when osimertinib was administered prior to PD-(L)1 blockade (11). This observation may indicate an extended functional half-life of PD-1 or PD-L1 inhibitors, suggesting that sequential administration could resemble concurrent use, particularly if immunotherapy is administered at the outset.

There are limitations in our case report. First, as a single-case report, the findings lack generalizability, making it challenging to speculate on the likelihood of this combination therapy inducing severe pneumonia. Although the clinical application of this combination therapy may be exceedingly rare, it nonetheless serves as a significant reminder of the potential risks involved. Second, during the course of methylprednisolone therapy for this patient, we did not administer any prophylactic treatment for PJP. A meta-analysis of randomized trials indicates that prophylaxis with TMP/SMX is warranted for non-human immunodeficiency virus (HIV) immunocompromised patients when the risk of PJP exceeds 6% (12). Additionally, for cancer patients receiving an equivalent of at least 20 mg of prednisone per day for more than four weeks, oral prophylaxis against PJP is recommended (13). Had preventive measures been implemented, it was possible that the patient’s prognosis could have improved, potentially avoiding mortality.

We propose that individuals undergoing immunotherapy in combination with EGFR-TKI may be at an increased risk of developing pneumonitis, potentially leading to a greater demand for corticosteroids. Furthermore, in instances of pneumonitis, these specific treatment contexts might necessitate more aggressive or prolonged immunosuppressive strategies due to the potential coexistence of two distinct types of toxicity-immune-related pneumonitis and EGFR-TKI-related pneumonitis-which could exacerbate symptom severity. In scenarios involving extended immunosuppression, especially in high-risk situations where two therapies known to induce pneumonitis are administered, it is imperative to consider the implementation of PJP prophylaxis.

## 4 Conclusion

Combinations of immunotherapy and Erlotinib should be avoided in routine clinical practice for patients with pancreatic cancer. Furthermore, we emphasize the necessity of careful consideration when selecting the initial treatment for patients with advanced pancreatic cancer. We advise exercising caution when contemplating the use of Erlotinib in patients who have recently undergone PD(L)-1 blockade therapy.

## Data Availability

The original contributions presented in the study are included in the article/supplementary material. Further inquiries can be directed to the corresponding author.
